# Pediatric cardiac arrest registries and survival outcomes: A European study

**DOI:** 10.1016/j.resplu.2025.100902

**Published:** 2025-02-11

**Authors:** Franziska Markel, Jana Djakow, Dominique Biarent, Nieves de Lucas, Jimena del Castillo, Sophie Skellett, Nigel M. Turner, Corinne M.P. Buysse, Kasper G. Lauridsen

**Affiliations:** a Department of Congenital Heart Disease – Pediatric Cardiology, Deutsches Herzzentrum der Charité, Augustenburger Platz 1, 13353 Berlin, Germany; b Department of Pediatric Pneumonology, Immunology and Intensive Care, Charité University Medicine Berlin, Augustenburger Platz 1, 13353 Berlin, Germany; c Heart Center Leipzig, University Hospital Leipzig, Strümpellstraße 39, 04289 Leipzig, Germany; d Paediatric Intensive Care Unit, NH Hospital, K Nemocnici 1106/14, 268 01 Hořovice, Czech Republic; e Paediatric Anaesthesiology and Intensive Care Medicine, University Hospital Brno, Medical Faculty of Masaryk University, Černopolní 212/9, 662 63 Brno, Czech Republic; f Paediatric Intensive Care & Emergency Department, Hôpital Universitaire des Enfants, Université Libre de Bruxelles, Avenue Jean-Joseph Crocq 7, 1020 Brussels, Belgium; g SAMUR – Protección Civil, Rda. de las Provincias, 7, Moncloa – Aravaca, 28011 Madrid, Spain; h Pediatric Intensive Care Department, Gregorio Marañon University Hospital, Calle de O’Donnell, 48, Retiro, 28009 Madrid, Spain; i Health Research Institute of the Gregorio Marañón Hospital, Madrid, Spain; j Pediatric Intensive Care Department, Great Ormond Street Hospital, Guilford St, London WC1N 3BH, United Kingdom; k Medical Education, University Medical Center, Heidelberglaan 100, 3584 CX Utrecht, the Netherlands; l Department of Neonatal and Pediatric Intensive Care, Division of Pediatric Intensive Care, Erasmus MC – Sophia Children’s Hospital, Dr. Molewaterplein 60, 3015 GJ Rotterdam, the Netherlands; m Department of Medicine, Randers Regional Hospital, Skovlyvej 15, 8930 Randers, Denmark; n Department of Anesthesiology and Critical Care Medicine, Children’s Hospital of Philadelphia, S 34th St &, Civic Center Blvd, Philadelphia, PA 19104, United States; o Research Center for Emergency Medicine, Aarhus University Hospital, Palle Juul-Jensens Blvd. 161, 8200 Aarhus, Denmark

**Keywords:** Pediatric cardiac arrest, Cardiac arrest registries, Outcomes, Epidemiology

## Abstract

**Objective:**

The epidemiology of pediatric cardiac arrest in Europe is largely unknown. We aimed to characterize pediatric cardiac arrest registries and obtain the first survival outcome data on pediatric cardiac arrest in Europe.

**Design:**

This is a prospective multinational survey.

**Setting:**

We surveyed all 53 countries in Europe asking about: the existence registries for pediatric out-of-hospital cardiac arrest (pOHCA) and/or in-hospital cardiac arrest (pIHCA)), the data collected, and the structure of the registries. Subsequently, we investigated outcomes (number of pOHCA/pIHCA since start of the registry, return of spontaneous circulation (ROSC), survival to hospital discharge/30-day survival) from the countries with active registries.

**Patients and interventions:**

We obtained information from 33 countries including 25 of the 27 European Union states.

**Measurements and main results:**

Thirteen countries (39%) have an ongoing pediatric cardiac arrest registry (pOHCA: 11 countries, pIHCA: 8 countries). All use the Utstein template for data collection. Five countries (15%) collect data about CPR quality. Eleven countries (33%) expressed interest in European collaboration on registry data. Overall, 13 countries reported data on outcomes from a total of 17,708 pOHCAs and 2,743 pIHCAs. The ROSC rate after pOHCA ranges from 10% to 72% as compared to 60% to 72% after pIHCA. Survival to hospital discharge ranges from 16% to 39% after pOHCA as compared to 32% to 57% after pIHCA.

**Conclusions:**

Less than 40% of the European countries have a pOHCA and/or pIHCA registry, reporting a wide variety in survival rates, especially after pOHCA. More systematic data collection is needed to identify the real incidence and outcomes from pediatric cardiac arrest, ideally through a joint European registry.

## Highlights

• Less than 40% of European countries have a pediatric cardiac arrest registry.
• Epidemiology of paediatric cardiac arrest remains challenging.
• Outcomes, especially after pediatric out-of-hospital cardiac arrest, differ greatly.
• Most countries are interested in a future collaboration.

## Introduction

Cardiac arrest is the most life-threatening condition in children and will result in death unless high-quality cardiopulmonary resuscitation (CPR) is provided. There are over 15,000 pediatric in-hospital cardiac arrests (pIHCA)^1^ and >20,000 pediatric out-of-hospital cardiac arrests (pOHCA)^2^, ^3^ each year in the US. Although Europe has a much larger population of more than 740,000,000 inhabitants, data on pediatric cardiac arrest for the continent are lacking.^4^, ^5^ There are few multicenter and single-country studies of pIHCA and pOHCA, and relatively little is known about the epidemiology of pediatric cardiac arrest in Europe.^6^, ^7^, ^8^

Pediatric cardiac arrest is a rare event but with many life-years at stake.^9^ Insufficient oxygenation and perfusion during cardiac arrest can lead to neurological injury that may result in anything from decreased school participation to permanently impaired daily living or even a vegetative state.^10^, ^11^ This has implications for healthcare economics and it is why the registry data are important to generate knowledge on outcomes of pediatric cardiac arrest patients. Registries can also provide data to inform initiatives on prevention of pediatric cardiac arrest and the quality of care improvement.^12^ For adult cardiac arrest, registries have provided essential information for international guidelines, clinical trials, and quality improvement initiatives.^13^ Accordingly, the European Resuscitation Council (ERC) and the International Liaison Committee on Resuscitation (ILCOR) recommend collecting data on cardiac arrest.^12^, ^14^ Guidance on which data to collect is given by the Utstein template and the pediatric core outcome set for cardiac arrest of ILCOR.^15^, ^16^, ^17^ To date, it is unknown how many countries in Europe follow these recommendations for pediatric cardiac arrest although numerous adult OHCA registries have provided invaluable data for decades. ^18^.

Therefore, the ERC Pediatric Life Support Science and Education Committee and the Young ERC considered it important to explore the current data collection on pediatric cardiac arrest in Europe and explore the opportunities for a future European registry collaboration.

As a step towards this, we aimed to characterize pediatric cardiac arrest registries in Europe and obtain the first data on survival outcomes after pOHCA and pIHCA in Europe.

## Methods

This is a trans-European cross-sectional study. We surveyed European countries for multicenter, regional, or national pediatric cardiac arrest registries using the REDCap survey tool (hosted by Aarhus University, Denmark).^19^ Under European legislation, ethical approval was not required for this survey-based study.

Using the World Health Organizatiońs definition of Europe, we identified European countries with a national resuscitation council (NRC).^20^ We asked the chair of the NRC for a suitable reference person to answer our survey. If no information could be gained via this method, or if there was no NRC, we contacted national course leaders of the European Pediatric Advanced Life Support Course (EPALS) or persons involved in relevant international pediatric societies within the country. The identified expert was asked whether he/she felt able to answer the questionnaire or to identify another person who could do this. We sent out a maximum of three reminder emails between July 2022 and January 2023 to this expert. The experts were contacted between January and November 2023 for a follow-up survey regarding survival outcomes.

### Data collection

The initial questionnaire was written in English and was divided into three parts: (A) 25 questions on registries in general, (B) 18 questions specifically relating to pOHCA, and (C) 25 questions on pIHCA. All three parts asked about the following characteristics of the registry: start date, current recruitment, duration of data collection, scope (e.g. national or regional, embedded in an adult registry, international collaboration), inclusion and exclusion criteria, directorship, funding, responsible person for data entry, type of electronic database, utilization of Utstein template and data variables collected. The general part additionally addressed the following topics: involvement in data collection, the role of the participant in relation to the registry, the combination of pOHCA and pIHCA in one registry, and interest in participation in a common European registry. The initial questionnaire can be seen in Appendix 1.

A follow-up questionnaire was sent to countries with an existing pediatric cardiac arrest registry and consisted of questions on: the total number of pOHCA and pIHCA since registry start, the rate of return of spontaneous circulation (ROSC), and rate of survival until hospital discharge or 30-day-survival after pOHCA and pIHCA respectively, as well as the duration of data collection.

The questionnaires were developed by members of the Young ERC and the ERC Pediatric Life Support Science and Education Committee. The survey was piloted and assessed for face validity by 5 ERC Pediatric Life Support Science and Education Committee members in July 2022.

### Data analysis

We used descriptive statistics. Binary data are reported as numbers (percentage).

## Results

**Fig. 1:**
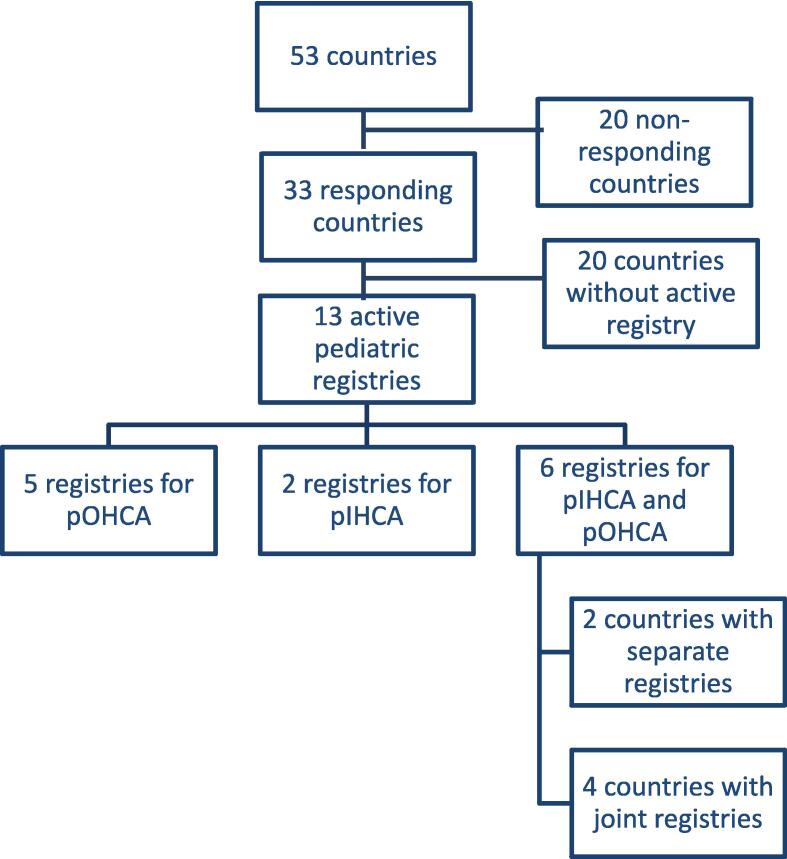
Flowchart of the responding and non-responding countries within Europe.

Of the 53 European countries surveyed, 33 responded (62%) including 25 out of 27 countries in the European Union (93%) (Fig. 1). Overall, 26 responses (79%) were obtained from national resuscitation councils and 7 responses (21%) were obtained from other sources (e.g. national course leaders). Of the 33 responding countries, 27 countries have an existing national resuscitation council as compared to only 4 among the 20 non-responding countries.

### Pediatric cardiac arrest registries in Europe

**Fig. 2:**
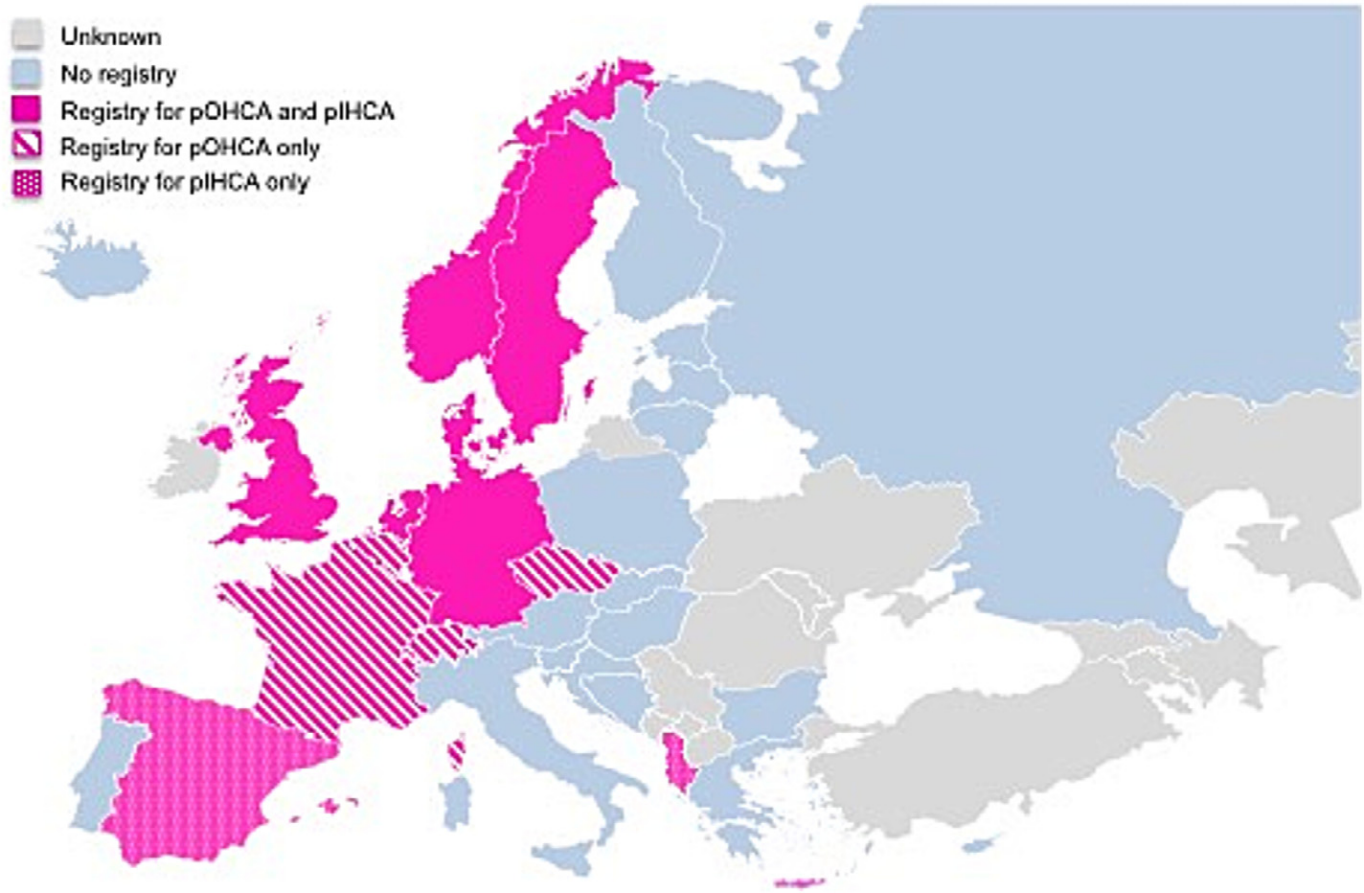
Of the 53 European countries included in the survey, 13 have an active paediatric cardiac arrest registry (pink). Countries with pOHCA registries are presented in pink with stripes, countries with pIHCA registries are presented in pink with dots, and countries with pOHCA and pIHCA registries are presented in plain pink. Countries presented in light blue did not have an active paediatric cardiac arrest registry at the time of the survey. Countries presented in grey have an unknown status. (For interpretation of the references to colour in this figure legend, the reader is referred to the web version of this article.)

Twenty countries (61%) do not have a registry (Fig. 2). Thirteen countries have an ongoing pediatric cardiac arrest registry: Six countries (18%) have a registry for both pOHCA and pIHCA (2 countries have separate registries; 4 countries collect the data in one registry, in three of these countries the data are embedded in an adult registry). Five countries (15%) collect data for pOHCA only, and 2 countries (6%) collect data for pIHCA only (Fig. 1, Fig. 2). Ten of these countries have nationwide coverage of the registry.

Some differences in inclusion- and exclusion criteria exist among the registries. Seven registries include events with bradycardia and poor perfusion, two registries include all events where ventilation or chest compressions are started (including respiratory arrests), and one registry includes pulseless cardiac arrests only. Three registries exclude patients over 16 years old and one registry excludes neonates below 30 days of age. Two registries exclude neonatal resuscitations and one excludes patients previously on support with extracorporeal membrane oxygenation.

Data on the directorship, funding, responsible person for data entry, and type of electronic database are shown in Appendix 2.

### Data collection

All countries use the Utstein template for their registries (Table 1).

**Table 1:** Variables collected in pediatric cardiac arrest registries. Data are reported as numbers (percent). PCPC = Pediatric Cerebral Performance Category, CPR = Cardiopulmonary Resuscitation, pOHCA = pediatric out-of-hospital cardiac arrest, pIHCA = pediatric in-hospital cardiac arrest.

	Pediatric Out-of-Hospital cardiac arrest registries	Pediatric In-Hospital-cardiac arrest registries
Variables	Total numbers (Percentage of pOHCA registries %)	Total numbers (Percentage of pIHCA registries %)
Use Utstein definitions	11 (100%)	8 (100%)
Age	11 (100%)	8 (100%)
Sex	11 (100%)	8 (100%)
Weight	4 (36%)	3 (38%)
Race/Ethnicity	2 (18%)	2 (25%)
Conditions/comorbidities	4 (36%)	4 (50%)
Illness category	5 (45%)	6 (75%)
Cause of cardiac arrest	10 (91%)	7 (86%)
Interventions prior to cardiac arrest	6 (55%)	4 (50%)
Baseline PCPC	3 (27%)	3 (38%)
Time of arrest	11 (100%)	7 (86%)
Site of arrest	11 (100%)	8 (100%)
Monitored arrest	5 (45%)	6 (75%)
Witnessed arrest	11 (100%)	7 (86%)
Emergency call time	11 (100%)	7 (86%)
Begin and end of CPR	11 (100%)	7 (86%)
Rhythm	11 (100%)	8 (100%)
Time to first rhythm check	8 (73%)	6 (75%)
Number of shocks	9 (82%)	6 (75%)
Time to first adrenaline	7 (64%)	5 (63%)
Number of adrenaline doses	7 (64%)	5 (63%)
CPR hemodynamics	3 (27%)	4 (50%)
CPR quality metrics	3 (27%)	3 (38%)
Post-resuscitation-care	7 (64%)	5 (63%)

**Fig. 3:**
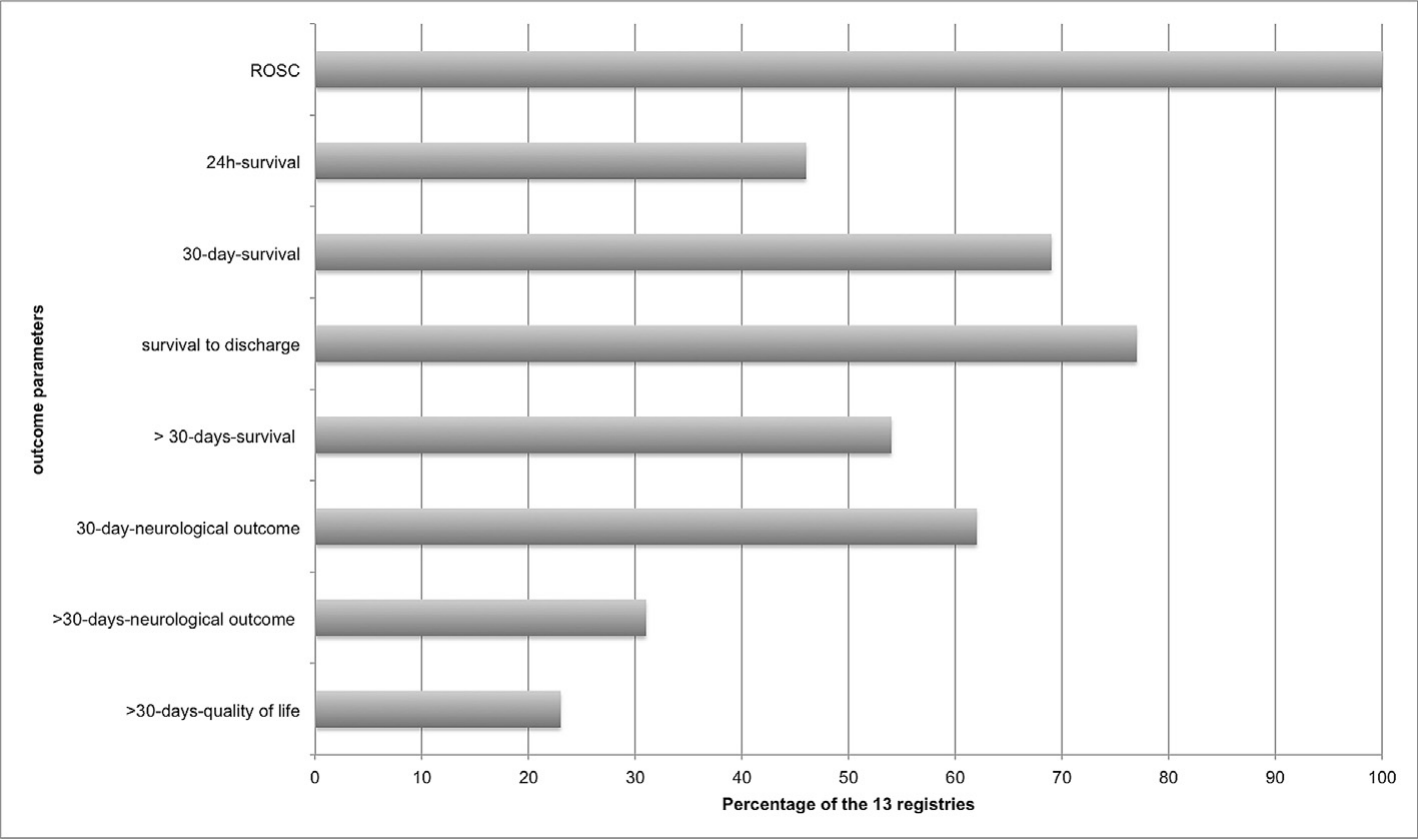
Outcome parameters that are collected by the 13 European registries (%).

There are some differences in the outcomes measured: 13 countries (39% of responding countries) collect data on ROSC, 10 countries (30%) collect data on survival to hospital discharge, 9 countries (27%) collect data on the 30-day survival and 8 countries (24%) collect data on the neurological outcome at discharge or after 30 days. Seven countries collect data on long-term outcomes beyond 30 days after the event, including survival (seven countries), neurological outcome (four countries) and quality of life (3 countries), as shown in Fig. 3. Overall, 5 countries (15%) collect data on CPR quality. Eleven countries (33%) expressed interest in participating in a common European registry.

### Pediatric cardiac arrest outcome data

Thirteen countries (100%) answered the follow-up survey on pediatric cardiac arrest data. The total number of pediatric cardiac arrests collected from each registry range from 20 to 5,796. Countries reported data on outcomes from a total of 17,708 pOHCAs and 2,743 pIHCAs (median duration of data collection pOHCA registry 11 years (quartiles 0.1–0.75: 6–21 years); median duration of data collection pIHCA registry 12 years (quartiles 0.1–0.75: 5–16 years)). After pOHCA the ROSC rate ranged from 10% to 72% and survival to hospital discharge or 30-day survival ranged from 5% to 39% (Table 2a).

**Table 2a:** Numbers of pediatric out-of-hospital cardiac arrests and survival outcomes from 11 European registries. *The country reports a large degree of data missing why caution should be taken when interpreting data. **ROSC to hospital admission, ***2002–2023; regional registry, covering 25% of The Netherlands, since 2023; nationwide registry.

pOHCA outcomes						
Country	Registry start	Registry design	pOHCÁs since registry start (n)	ROSC rate (%)	Survival to discharge (%)	30-day survival (%)
Netherlands	2002	***	400	72	39	39
Czech Republic	2017	regional	47	53	21	18
Switzerland	2018	national	445	39	16*	N/A
Germany	2007	national	1740	43	N/A	5*
Sweden	1990	national	5796	35*	N/A	3
Norway	2016	national	308	33	N/A	21
France	N/A	national	1500	20	N/A	5
Belgium	2017	national	154	37	20	15
Denmark	2001	national	1983	35	N/A	23
Great Britain and Northern Ireland	2011	national	5315	20**	N/A	9
Crimea	2018	regional	20	10	N/A	N/A

In comparison, after pIHCA the ROSC rate ranged from 60% to 71% and survival to hospital discharge or 30-day survival ranged from 32% to 58% (Table 2b).

**Table 2b:** Numbers of pediatric in-hospital cardiac arrests and survival outcomes from 8 European registries. *2002–2023; regional registry, covering 25% of The Netherlands, since 2023; nationwide registry.

pIHCA outcomes						
Country	Registry start	Registry design	pIHCÁs since registry start	ROSC rate (%)	Survival to discharge (%)	30-day survival (%)
Netherlands	2002	*	200	N/A	57	N/A
Spain	2020	national	91	60	32	N/A
Germany	2007	national	N/A	N/A	N/A	N/A
Sweden	2018	national	382	47	N/A	58
Denmark	2013	national	135	71	N/A	53
Great Britain and Northern Ireland	2011	national	1935	68	53	N/A

## Discussion

This is the first study to report overall epidemiological data on pediatric cardiac arrest in Europe. We identified 15 different pediatric cardiac arrest registries from 13 countries with large variations in outcomes for pOHCA and pIHCA between European countries.

Compared to a previous literature review on pediatric cardiac arrest registries, this appears to be a positive trend in the number of registries but it must be emphasized that 20 of the responding European countries still do not have a registry.^21^ Notably, we identified a larger number of registries for pOHCA than for pIHCA. This is similar for adult cardiac arrest registries of which more are established, and more randomized trials are conducted for, OHCA as compared to IHCA.^22^ Ten of the pOHCA registries are embedded in an adult registry. Utilization of the established infrastructure of an adult OHCA registry offers obvious benefits but may also carry the disadvantage of collecting data on the same parameters for pediatric and adult cardiac arrests even though the parameters of interest may differ between age-groups.^23^.

All registries make use of the Utstein template. However, only 2 registries collect all the suggested outcomes of the *pediatric core outcome set after cardiac arrest* (P-COSCA).^16^ The P-COSCA is intended to inform clinical trials on reporting of pediatric cardiac arrest outcomes and follow-up data on quality of life may still be relevant in registries. As the long-term consequences after pOHCA and pIHCA, such as survival beyond 30-days, long-term neurological outcome, and quality of life are of particular importance to patients and families, inclusion of such data in registries should be encouraged.

Our data demonstrate very large differences in outcomes, especially after pOHCA where ROSC rates ranged from 10% to 72%. In comparison, Gräsner et al. reported ROSC rates from 9% to 50% for adult OHCA in 27 European countries, while survival to hospital discharge following adult OHCA has been reported to vary from 3% to 20% globally.^18^, ^24^ This compares to reported ROSC rates of 30% in Japan and 16% in the US and reported survival with favorable neurological outcome of 9.9% in Japan and 8.7% in the US.^3^, ^25^

The differences between countries in survival from pIHCA are smaller than for pOHCA but still considerable. Data from the US report ROSC rates after pIHCA of 78–90% and survival to hospital discharge of 32–40%.^26^

The reasons for the disparities in survival outcomes after pediatric out-of-hospital and in-hospital cardiac arrest are unknown. Possible causes might include registration factors, including varying coverage of the registries, varying time period of data collection, missing data on survival outcomes, a different interpretation of cardiac arrest and therefore selective reporting (e.g. inclusion of bradycardia with poor perfusion or pulseless events only).

We found that the completeness of the registry data varies greatly between countries and several countries report missing data on the majority of patients. Although the reasons for this are unknown, multiple factors may possibly affect data completeness including design and infrastructure of the registry, voluntary participation, funding or lack of attention to reporting pediatric cardiac arrest in registries that were originally designed to cover adult cardiac arrests only.

Even more challenging is the interpretation of the influence of differences in healthcare systems and efficacy in the chain of survival among all countries.^27^ Gräsner et al. explained the disparity in adult OHCA survival as the result of different EMS coverage and CPR practices.^18^ CPR training of laypeople is associated with improved bystander CPR rates and survival outcomes after OHCA in adults,^28^, ^29^ but the training differs greatly among European countries.^30^ Notably, registry data show that the majority of pOHCA in smaller children occur in private homes, whereas cardiac arrests for older children and adolescents often occur outside of home.^31^, ^32^ Thus, targeted training for teachers and caregivers of children and adolescents may be considered and such initiatives should ideally be investigated in future research.

In addition, other system factors such as AED coverage, implementation of first responder systems, Emergency Medical Team (EMT) coverage, ambulance arrival times, transport times to hospital and hospital density or centralization of PICÚs may also play a role in the differing survival outcomes.^18^, ^24^ Hospital factors may include CPR training, rapid response systems to prevent cardiac arrest, cardiac arrest teams to improve CPR quality, quality improvement initiatives including debriefings and specialized intensive care units to provide optimal post-arrest care including ECMO utilization. 13, 33, 34, 35, 36, 37, 38, 39, 40

This is the first study to investigate the epidemiology of pediatric cardiac arrest in Europe. Yet, differences in registries, variable coverage and missing data make the picture incomplete. To overcome implementation barriers^35^, pediatric cardiac arrest registries may need financial and political support to establish a robust infrastructure which can generate reliable data for research.

The majority of countries stated an interest in a European pediatric cardiac arrest registry. Initiating a joint pediatric cardiac arrest registry would have several challenges including joint definitions on in- and exclusion criteria and outcomes. However, a joint European pediatric cardiac arrest registry could provide an opportunity to investigate the epidemiology of pediatric cardiac arrest in detail as well as explore the underlying reasons for differences in outcomes and the impact of quality improvement initiatives and quality of care on survival outcomes. Multinational collaboration may also enable studies on training initiatives in and outside of hospitals, bystander CPR rates and survival outcomes that are otherwise difficult to conduct. An infrastructure for systematic follow-up strategies for survivors of pediatric cardiac arrest may help to improve long-term neurological function and consequently quality of life. Use of a single large cardiac arrest registry in the US has led to hundreds of scientific publications informing international guidelines, local benchmarking fostering quality improvement initiatives, and increased public awareness with gradually increasing survival outcomes over time.^26^, ^41^, ^42^

This study reports on the existence or absence of pediatric cardiac arrest registries in 33 European countries, which include the majority of the population of Europe. However, data could not be obtained from 20 countries. The list of missing countries includes those of a relatively small population and countries in low- and middle-income settings, which may lack the necessary infrastructure for a registry, including a national resuscitation council. Consequently, it is our belief that the present study provides a reasonable overview of the registries, although the proportion of European countries without a registry is likely to be higher than that reported in this study.

### Limitations

We cannot infer on the exact coverage and completeness of each registry and the true incidence of pOHCA and pIHCA is therefore unknown. Since we do not have insight into individual patient data, we cannot draw specific conclusions about outcomes. There were some variations in inclusion and exclusion criteria relating to minimal inclusion age of 30 days and maximum age of 16 years vs. 18 years of age and some countries including bradycardia with poor perfusion. Moreover, we cannot rule out any differences in local practices for inclusion in each registry that may not be described as official exclusion criteria per se, e.g. cases where healthcare professionals terminate CPR or decide not to start CPR due to futility. Finally, there may be large variations in the amount of missing data on survival outcomes between countries, so data on survival in each country should be interpreted with caution and survival outcomes cannot be directly compared.

## Conclusion

Fewer than 40% of European countries have registries for pOHCA and/or pIHCA and most registries collect data on pOHCA. Survival rates vary considerably, particularly after pOHCA. More data with national coverage are needed to determine the real incidence of pediatric cardiac arrest, ideally through a joint, European registry with standardized data collection.

## CRediT authorship contribution statement

**Franziska Markel:** Writing – review & editing, Writing – original draft, Visualization, Project administration, Methodology, Investigation, Formal analysis, Data curation, Conceptualization. **Jana Djakow:** Writing – review & editing, Investigation, Conceptualization. **Dominique Biarent:** Writing – review & editing, Investigation, Conceptualization. **Nieves de Lucas:** Writing – review & editing, Investigation, Conceptualization. **Jimena del Castillo:** Writing – review & editing, Investigation, Conceptualization. **Sophie Skellett:** Writing – review & editing, Investigation, Conceptualization. **Nigel M. Turner:** Writing – review & editing, Investigation, Conceptualization. **Corinne M.P. Buysse:** Writing – review & editing, Investigation, Conceptualization. **Kasper G. Lauridsen:** Writing – review & editing, Supervision, Resources, Project administration, Methodology, Investigation, Formal analysis, Conceptualization.

## Declaration of competing interest

The authors declare the following financial interests/personal relationships which may be considered as potential competing interests: “All authors are members of the Pediatric Life Support Science and Education Committee of the European Resuscitation Council. Jana Djakow, Jimena del Castillo, and Kasper G. Lauridsen are the International Liaison Committee on Resuscitation Task Force members. Kasper G. Lauridsen has current research project grants from the Central Denmark Region, Independent Research Fund Denmark, and the Laerdal Foundation. Franziska Markel receives current research grants from the “Stiftung Kinderherzen” and the “German Association for Pediatric Cardiology”. The views expressed are those of the authors and do not necessarily reflect those of the ERC, the national resuscitation registries, or their funders. The authors state that there is no other declaration of interest.”.
