# Supplementary material for: Pediatric cardiac arrest registries and survival outcomes: A European study
Source: Resusc Plus. 2025 Feb 11;22:100902. doi: 10.1016/j.resplu.2025.100902 (PMC11880730; doi:10.1016/j.resplu.2025.100902)
Supplement: Supplementary Appendix 1 [file mmc1.pdf]

# Pediatric cardiac arrest registry survey

This questionnaire is about pediatric cardiac arrest registries (in-hospital and out-of-hospital). The questionnaire takes 5-10 minutes to complete. We kindly ask for your time to answer these questions as this will be an important foundation for future international work on cardiac arrest registries aiming to improve survival outcomes for children suffering a cardiac arrest.

Thank you for your time!

The European Resuscitation Council Paediatric Life Support Science and Education Committee

| | |
|---|---|
| Which country do you represent? | ◯ Albania<br>◯ Andorra<br>◯ Armenia<br>◯ Austria<br>◯ Azerbaijan<br>◯ Belarus<br>◯ Belgium<br>◯ Bosnia and Herzegovina<br>◯ Bulgaria<br>◯ Croatia<br>◯ Cyprus<br>◯ Czech Republic<br>◯ Denmark<br>◯ Estonia<br>◯ Finland<br>◯ France<br>◯ Georgia<br>◯ Germany<br>◯ Greece<br>◯ Hungary<br>◯ Iceland<br>◯ Ireland<br>◯ Israel<br>◯ Italy<br>◯ Kazakhstan<br>◯ Kyrgyzstan<br>◯ Latvia<br>◯ Lithuania<br>◯ Luxembourg<br>◯ Malta<br>◯ Monaco<br>◯ Montenegro<br>◯ Netherlands<br>◯ North Macedonia<br>◯ Norway<br>◯ Poland<br>◯ Portugal<br>◯ Republic of Moldova<br>◯ Romania<br>◯ Russian Federation<br>◯ San Marino<br>◯ Serbia<br>◯ Slovakia<br>◯ Slovenia<br>◯ Spain<br>◯ Sweden<br>◯ Switzerland<br>◯ Tajikistan<br>◯ Turkey<br>◯ Turkmenistan<br>◯ Ukraine<br>◯ United Kingdom of Great Britain and Northern Ireland<br>◯ Crimea |
| Does your country have a registry collecting data on pediatric cardiac arrest? | ◯ Yes, for pediatric In-hospital cardiac arrest (p-IHCA)<br>◯ Yes, for out-of-hospital cardiac arrest (p-OHCA)<br>◯ Yes, for both<br>◯ I don´t know<br>◯ No, we do not have a registry |

| | |
|---|---|
| What is the name of the registry/ registries? | __________________________________ |
| Is this pediatric registry active/currently recruiting patients? | ☐ Yes.<br>☐ No.<br>☐ I don´t know. |
| When did the registry start? | ___________________________ |
| For how many years has the registry been collecting data? | ___________________________ |
| Do you/your work place participate in the data collection for the registry? | ◯ Yes, we collect data for the registry.<br>◯ No, we don´t collect data for the registry.<br>◯ I don´t know. |
| What is your role concerning the registry? | ☐ Member of the national resuscitation council<br>☐ Member of an international resuscitation council<br>☐ Member of a regional resuscitation initiative<br>☐ Member of a scientific society<br>☐ Chair/member of the board/steering committee for the registry<br>☐ an administrative worker for the registry<br>☐ Other |
| Please specify "other". | ___________________________ |
| Is it two separate registries or one combined registry? | ◯ It is one registry covering both pediatric in-hospital cardiac arrest (p-IHCA) and pediatric out-of-hospital cardiac arrest (p-OHCA)<br>◯ We have two separate registries.<br>◯ I don´t know. |
| Is this registry collecting information on all pediatric patients? | ◯ Yes, all pediatric patients are included.<br>◯ No, only pediatric patients in the intensive care unit/emergency care are included.<br>◯ I don´t know.<br>◯ Other |
| Please specify "other". | __________________________________ |
| Is your registry embedded in an adult registry or is it separate? | ◯ Yes, it is embedded in an adult registry.<br>◯ No, it is separate.<br>◯ I don´t know.<br>◯ Other |
| Please specify "other". | __________________________________ |

| | |
|---|---|
| Is it a national/multi-institutional registry? | ◯ Yes, it is a national covering all parts of the country.<br>◯ Yes, it is a national/multi-institutional registry, but not all hospitals are participating.<br>◯ No, it is a regional registry.<br>◯ I don´t know.<br>◯ Other |
| Please specify "other". | __________________________________ |
| Is the registry part of an international collaboration of registries? | ◯ Yes, it is part of an international collaboration.<br>◯ No, it is not part of an international collaboration.<br>◯ I don´t know.<br>◯ Other |
| Please specify "other". | __________________________________ |
| Who is the leader of the registry? | ◯ University<br>◯ Hospital<br>◯ Governmental department<br>◯ Medical Society<br>◯ Emergency Medical Services<br>◯ Independent Institution<br>◯ Other<br>◯ I don´t know. |
| Please specify "other". | __________________________________ |
| How is the registry funded? | ◯ Funding from national governmental organizations<br>◯ Funding from private organizations<br>◯ Funding from science societies<br>◯ Other<br>◯ No funding<br>◯ I don´t know. |
| Please specify "other". | __________________________________ |
| Who is responsible for entering the data into the online database? | ◯ study nurse/researcher/medical student/other during paid work time<br>◯ researcher/medical student/other during free time, unpaid<br>◯ I don´t know<br>◯ other |
| Please specify "other". | __________________________________ |

| | |
|---|---|
| What electronic database do you use? | ◯ REDCap<br>◯ Castor<br>◯ OpenClinica<br>◯ other<br>◯ do not know |
| Please specify "other". | __________________________________ |
| Do you include threatening cardiac arrests/apparent life-threatening events? | ◯ Yes, we include all emergencies leading to an emergency call.<br>◯ Yes, we include all events where ventilation or compressions are started (e.g. respiratory arrests).<br>◯ We include all events where chest compressions are started, including bradycardia with poor perfusion.<br>◯ We include pulseless cardiac arrests where CPR is started only.<br>◯ I don´t know.<br>◯ Other |
| Please specify "other". | __________________________________________ |
| What is the minimum age of patients included in the registry (in days)? | __________________________________ |
| What is the maximum age of patients included in the registry (years)? | __________________________________ |
| Please describe any inclusion or exclusion criteria other than age and add any other comments you have relating to in- or exclusion criteria. | __________________________________________ |
| Are you using the Utstein template for your data input? | ◯ Yes, we are using the Utstein template for our data input.<br>◯ No, we are using different parameters.<br>◯ I don´t know. |

| | |
|---|---|
| Which of the following parameters are included in your registry? | ☐ Age<br>☐ sex<br>☐ weight<br>☐ racial and ethnic categories<br>☐ pre-existing conditions/ comorbidities<br>☐ illness category<br>☐ cause of arrest<br>☐ interventions in place prior to arrest (e.g. tracheal tube)<br>☐ baseline pediatric cerebral performance category<br>☐ time of the arrest<br>☐ site of the arrest<br>☐ monitored arrest<br>☐ witnessed arrest<br>☐ emergency call (time)<br>☐ begin and end of CPR (or CPR duration)<br>☐ presenting rhythm (shockable/non-shockable)<br>☐ time to first rhythm check<br>☐ number of shocks<br>☐ time to first adrenaline<br>☐ number of adrenaline doses<br>☐ CPR hemodynamics (ET-CO2 and/ or a-line)<br>☐ CPR quality metrics (compression rate, depth, pauses, and/ or ventilation rate)<br>☐ post-resuscitation care (e.g. temperature management, glucose, oxygenation, neuroprognostication)<br>☐ I don´t know.<br>☐ Other |
| Please specify "other". | __________________________________ |
| Which outcome data do you collect? | ☐ Return of spontaneous circulation (ROSC)<br>☐ 24 hour survival<br>☐ survival to hospital discharge<br>☐ survival to 30 days<br>☐ neurological outcome at discharge/ 30 days<br>☐ long-term survival beyond discharge/ 30 days<br>☐ long-term neurological outcome beyond discharge/ 30 days<br>☐ long-term quality of life beyond discharge/ 30 days<br>☐ I don´t know<br>☐ Other |
| Please specify "other". | __________________________________ |
| Would you be interested to discuss participation in a European joint registry? | ◯ Yes.<br>◯ No.<br>◯ I don´t know. |
| We like to contact you for a follow-up survey and an exchange of ideas. If you agree, or if you would like to recommend a more appropriate person from your country please leave your mail address. | ____________________________ |
| Please upload your data entry form, if possible. | |

**The following questions concern the pediatric OUT-OF-hospital cardiac arrest (p-OHCA) registry ONLY**

What is the name of the registry?

__________________________________

Is your pediatric out-of-hospital cardiac arrest (p-OHCA) registry active/currently recruiting patients?

◯ Yes.
◯ No.
◯ I don´t know.

When did your p-OHCA registry start?

__________________________________

For how many years has the p-OHCA registry been collecting data?

__________________________________

Is your p-OHCA registry embedded in an adult registry or is it separate?

◯ Yes, it is embedded in an adult registry
◯ No, it is separate
◯ I don´t know
◯ Other

Please specify "other".

__________________________________________

Is it a nationwide registry or covering parts of the country?

◯ Yes, it is a national registry, covering all parts of the country
◯ Yes, it is a national/ multi-institutional registry, but not all hospitals are participating
◯ No, it is a regional registry
◯ I don´t know
◯ Other

Please specify "other".

__________________________________________

Is the registry part of an international collaboration of registries?

◯ Yes, it is part of an international collaboration.
◯ No, it is not part of an international collaboration.
◯ I don´t know
◯ Other

Please specify "other".

__________________________________________

Who is the leader of the p-OHCA registry?

◯ University
◯ Hospital
◯ Governmental department
◯ Medical Society
◯ Emergency Medical Services
◯ Independent Institution
◯ Other
◯ I don´t know

| | |
|---|---|
| Please specify "other". | __________________________________ |
| How is the registry funded? | ◯ Funding from national governmental organizations<br>◯ Funding from private organizations<br>◯ Funding from science societies<br>◯ Other<br>◯ No funding<br>◯ I don´t know |
| Please specify "other". | __________________________________ |
| Who is responsible for entering the data into the online database? | ◯ study nurse/researcher/medical student/other during paid work time<br>◯ researcher/medical student/other during free time, unpaid<br>◯ I don´t know<br>◯ other |
| Please specify "other". | __________________________________ |
| What electronic database do you use? | ◯ REDCap<br>◯ Castor<br>◯ OpenClinica<br>◯ other |
| Please specify "other". | __________________________________ |
| Do you include threatening cardiac arrests/apparent life-threatening events or non-survival at scene (please mark all relevant answers)? | ☐ Yes, we include all emergencies leading to an emergency call.<br>☐ Yes, we include all events where ventilation or compressions are started (e.g. respiratory arrests).<br>☐ We include all events where chest compressions are started, including bradycardia with poor perfusion.<br>☐ We include pulseless cardiac arrests where CPR is started only.<br>☐ We include children with p-OHCA without ROSC and non-survival at scene.<br>☐ We do not include children with non-survival at scene.<br>☐ I don´t know.<br>☐ Other |
| Please specify "other". | __________________________________ |
| What is the minimum age of patients included in the registry (in days)? | __________________________________ |
| What is the maximum age of patients included in the registry (years)? | __________________________________ |

Please describe any inclusion or exclusion criteria other than age and add any other comments you have relating to in- or exclusion criteria.

__________________________________

Are you using the Utstein template for your data input?

- ◯ Yes, we are using the Utstein template for our data input.
- ◯ No, we are using different parameters.
- ◯ I don´t know.

Which of the following parameters are included in your pediatric out-of-hospital cardiac arrest registry?

- ☐ Age
- ☐ sex
- ☐ weight
- ☐ racial and ethnic categories
- ☐ pre-existing conditions/ comorbidities
- ☐ illness category
- ☐ cause of arrest
- ☐ interventions in place prior to arrest (e.g. tracheal tube)
- ☐ baseline pediatric cerebral performance category (PCPC)
- ☐ time of the arrest
- ☐ site of the arrest
- ☐ monitored arrest
- ☐ witnessed arrest
- ☐ emergency call (time)
- ☐ begin and end of CPR (or CPR duration)
- ☐ presenting rhythm (shockable/non-shockable)
- ☐ time to first rhythm check
- ☐ number of shocks
- ☐ time to first adrenaline
- ☐ number of adrenaline doses
- ☐ CPR hemodynamics (ET-CO2 and/ or a-line)
- ☐ CPR quality metrics (compression rate, depth, pauses, and/ or ventilation rate)
- ☐ post-resuscitation care (e.g. temperature management, glucose, oxygenation, neuroprognostication)
- ☐ I don´t know
- ☐ Other

Please specify "other".

__________________________________

Which outcome data do you collect in the pediatric out-of-hospital cardiac arrest registry?

- ☐ Return of spontaneous circulation (ROSC)
- ☐ 24 hour survival
- ☐ survival to hospital discharge
- ☐ survival to 30 days
- ☐ neurological outcome at discharge/ 30 days
- ☐ long-term survival beyond discharge/ 30 days
- ☐ long-term neurological outcome beyond discharge/ 30 days
- ☐ long-term Quality of life beyond discharge/ 30 days
- ☐ I don´t know
- ☐ Other

Please specify "other".

__________________________________

Please upload your data entry form, if possible.

**The following questions concern the pediatric IN-HOSPITAL cardiac arrest (p-IHCA) registry ONLY**

What is the name of the registry? ______________________________

Is your p-IHCA registry active/currently recruiting patients?
- ◯ Yes.
- ◯ No.
- ◯ I don´t know.

When did your p-IHCA registry start? ______________________________

For how many years has the p-IHCA registry been collecting data? ______________________________

Is your p-IHCA registry embedded in an adult registry or is it separate?
- ◯ Yes, it is embedded in an adult registry
- ◯ No, it is separate
- ◯ I don´t know
- ◯ Other

Please specify "other". _____________________________________

Is it a nationwide p-IHCA registry or covering parts of the country?
- ◯ Yes, it is a national registry, covering all parts of the country.
- ◯ Yes, it is a national/ multi-institutional registry, but not all hospitals are participating.
- ◯ No, it is a regional registry.
- ◯ I don´t know.
- ◯ Other

Please specify "other". _____________________________________

Is the registry part of an international collaboration of registries?
- ◯ Yes, it is part of an international collaboration.
- ◯ No, it is not part of an international collaboration.
- ◯ I don´t know.
- ◯ Other

Please specify "other". _____________________________________

Who is the leader of the p-IHCA registry?
- ◯ University
- ◯ Hospital
- ◯ Governmental department
- ◯ Medical Society
- ◯ Emergency Medical Services
- ◯ Independent Institution
- ◯ Other
- ◯ I don´t know

| | |
|---|---|
| Please specify "other". | __________________________________ |
| How is the registry funded? | ◯ Funding from national governmental organizations<br>◯ Funding from private organizations<br>◯ Funding from science societies<br>◯ Other<br>◯ No funding<br>◯ I don´t know |
| Please specify "other". | __________________________________ |
| Who is responsible for entering the data into the online database? | ◯ study nurse/researcher/medical student/other during paid work time<br>◯ researcher/medical student/other during free time, unpaid<br>◯ I don´t know<br>◯ other |
| Please specify "other". | ____________________________ |
| What electronic database do you use? | ◯ REDCap<br>◯ Castor<br>◯ OpenClinica<br>◯ other |
| Please specify "other". | ____________________________ |
| Do you include threatening cardiac arrests/apparent life-threatening events? | ◯ Yes, we include all emergencies leading to an emergency call.<br>◯ Yes, we include all events where ventilation or compressions are started (e.g. respiratory arrests).<br>◯ We include all events where chest compressions are started, including bradycardia with poor perfusion.<br>◯ We include pulseless cardiac arrests where CPR is started only.<br>◯ I don´t know.<br>◯ Other |
| Please specify "other". | __________________________________ |
| What is the minimum age of patients included in the registry (in days)? | ____________________________ |
| What is the maximum age of patients included in the registry (years)? | ____________________________ |
| Please describe any inclusion or exclusion criteria other than age and add any other comments you have relating to in- or exclusion criteria. | __________________________________ |

| | |
|---|---|
| Are you using the Utstein template for your data input? | ◯ Yes, we are using the Utstein template for our data input.<br>◯ No, we are using different parameters.<br>◯ I don´t know. |
| Which of the following parameters are included in your p-IHCA registry? | ☐ Age<br>☐ sex<br>☐ weight<br>☐ racial and ethnic categories<br>☐ pre-existing conditions/ comorbidities<br>☐ illness category<br>☐ cause of arrest<br>☐ interventions in place prior to arrest (e.g. tracheal tube)<br>☐ baseline pediatric cerebral performance category (PCPC)<br>☐ time of the arrest<br>☐ site of the arrest<br>☐ monitored arrest<br>☐ witnessed arrest<br>☐ emergency call (time)<br>☐ begin and end of CPR (or CPR duration)<br>☐ presenting rhythm (shockable/non-shockable)<br>☐ time to first rhythm check<br>☐ number of shocks<br>☐ time to first adrenaline<br>☐ number of adrenaline doses<br>☐ CPR hemodynamics (ET-CO2 and/ or a-line)<br>☐ CPR quality metrics (compression rate, depth, pauses, and/ or ventilation rate)<br>☐ post-resuscitation care (e.g. temperature management, glucose, oxygenation, neuroprognostication)<br>☐ I don´t know<br>☐ Other |
| Please specify "other". | __________________________________ |
| Which outcome data do you collect in the p-IHCA registry? | ☐ Return of spontaneous circulation<br>☐ 24 hour survival<br>☐ survival to hospital discharge<br>☐ survival to 30 days<br>☐ neurological outcome at discharge/ 30 days<br>☐ long-term survival beyond discharge/ 30 days<br>☐ long-term neurological outcome beyond discharge/ 30 days<br>☐ long-term quality of life beyond discharge/ 30 days<br>☐ I don´t know<br>☐ Other |
| Please specify "other". | __________________________________ |
| Please upload your data entry form, if possible. | |
