# Supplementary material for: Pediatric cardiac arrest registries and survival outcomes: A European study
Source: Resusc Plus. 2025 Feb 11;22:100902. doi: 10.1016/j.resplu.2025.100902 (PMC11880730; doi:10.1016/j.resplu.2025.100902)
Supplement: Supplementary Appendix 2 [file mmc2.docx]

**Appendix 2**

| **Directorship** | |
| --- | --- |
| Hospital | 3 |
| Emergency Medical Service | 2 |
| Governmental department | 2 |
| Independent institution | 3 |
| Medical society | 1 |
| Unknown | 2 |
| **Data entry by...** | |
| Study nurse/researcher/medical student/other during paid work time | 5 |
| Researcher/medical student/other during free time, unpaid | 4 |
| Other* | 2 |
| Unknown | 2 |
| **Electronic database** | |
| REDCap | 1 |
| Castor | 1 |
| Other** | 7 |
| Unknown | 4 |
| **Funding** | |
| Yes | 10 |
| No | 2 |
| Unknown | 1 |

Appendix 2: Data of pediatric cardiac arrest registries (n) on funding, responsible staff for data entry, directorship and type of electronic database. *All cases are entered into an offline database by a registrar (staff psychologist) and double checked by a researcher (physician - anesthesiologist) during paid work time.//The data is entered directly by the health care provider involved in the case. **Microsoft Access 2016, Microsoft Corporation, USA//Home made//Ad hoc created database and Utstein style compliant//Xolomon//National registry database for medical quality registries.
